# A systematic review on masticatory muscle activity of temporomandibular joint in patients with rheumatoid arthritis

**DOI:** 10.2340/aos.v84.44959

**Published:** 2025-12-15

**Authors:** Kaltrina Kryeziu, Mergime Prekazi Loxha, Besim Hajdari, Leminot Salihu, Mjellma Rexhepi, Rrezarta Alihajdaraj, David Stubljar, Andrej Starc

**Affiliations:** a University of Prishtina, Faculty of Medicine, Department of Maxillofacial Surgery, Pristina, Kosovo; b University of Prishtina, Faculty of Medicine, Department of Rheumatology, Pristina, Kosovo; c Clinical University Center of Kosova, Department of Maxillofacial Surgery, Pristina, Kosovo; d In-Medico, Department of Research and Development, Metlika, Slovenia; e Chair of Public Health, Faculty of Health Sciences, University of Ljubljana, Ljubljana, Slovenia

**Keywords:** masticatory muscles, quality of life, rheumatoid arthritis, systematic review, temporomandibular joint

## Abstract

Rheumatoid arthritis (RA) is a systemic autoimmune disorder characterized by chronic inflammation, primarily targeting joints. When it affects the temporomandibular joint (TMJ), it can significantly diminish the quality of life for individuals. Despite the increasing recognition of disorders involving TMJ and the importance of the stomatognathic system, there is a limited number of studies on masticatory muscle activity of TMJ in patients with RA and the changes in the function of these keychewing muscles. The objective of this study was to conduct a comprehensive systematic review of existing evidence concerning the functioning of major chewing muscles in patients with RA, specifically focusing on parameters such as chewing force. Findings from existing research indicate that RA patients exhibit higher prevalence of bone changes in the TMJ, including erosion, flattening, sclerosis, and osteophytes. These alterations are typically diagnosed through cone-beam computed tomography (CBCT). Results demonstrate that individuals with RA are more likely to experience bone changes in TMJ structures compared to those without RA. By synthesizing available data, this review aims to provide insights that can inform clinical management strategies and potentially improve outcomes for patients grappling with RA-related TMJ involvement. This article aims to review TMJ disorder in RA patients.

## Introduction

The temporomandibular joint (TMJ) is a synovial joint, which is positioned between the head of the mandible, the articular tubercle, and the mandibular fossa of the temporal bone [1]. It plays important roles in crucial processes such as chewing, speaking, and swallowing, which affect every part of society.

## Anatomy of the temporomandibular joint

TMJ is a ginglymoarthrodial joint, formed by the articulation between the mandibular condyle and the glenoid fossa of the temporal bone. An articular disc divides the joint into two separate synovial cavities, each with distinct movement patterns. The superior cavity, between the articular disc and the glenoid fossa, is responsible for gliding or translatory movements, while the inferior cavity, between the disc and the condyle, allows for rotary or hinge movements. The TMJ’s stability is maintained by three key ligaments: the temporomandibular, stylomandibular, and sphenomandibular ligaments. The muscles involved in temporomandibular disorders include the muscles of mastication: the temporalis, masseter, and medial and lateral pterygoids. The joint’s primary blood supply comes from the superficial temporal and maxillary branches of the external carotid artery, with additional contributions from the anterior tympanic, deep auricular, and ascending pharyngeal arteries. Sensory innervation is provided by the auriculotemporal and masseteric branches of the mandibular nerve (V3), which is a division of the trigeminal nerve [2].

## Disorders of the TMJ

Temporomandibular disorders (TMDs) are a group of conditions affecting the TMJ, the muscles that control jaw movement, and the surrounding tissues. These disorders can cause pain, restricted jaw movement, and audible clicking or popping sounds in the joint. TMDs can arise from various factors, including injury, arthritis, stress, or misalignment of the teeth or jaw. Symptoms can range from mild discomfort to severe pain and dysfunction, with women between the ages of 20 and 40 being more commonly affected. Treatment varies depending on the severity and underlying cause, often involving a combination of self-care, physical therapy, medications, or, in severe cases, surgery [2, 3].

TMDs can be presented as different conditions:

Derangement of the Condyle-Disc Complex

– Disc dislocation with reduction
– Disc dislocation without reduction
– Structural incompatibility with articular surfaces

Deviation in the form

– Adherences and adhesions
– Subluxation
– Luxation (dislocation)

Inflammatory disorders of the TMJ

– Synovitis/capsulitis
– Retrodiscitis
– Arthralgia
– Arthritis
– Osteoarthritis
– Osteoarthrosis
– Systemic arthritis

Chronic mandibular hypomobility

– Ankylosis
– Muscle contracture
– Coronoid process impedance

Growth disorders

– Agenesis (no growth)
– Hypoplasia (insufficient growth)
– Hyperplasia (excessive growth)
– Neoplasia (uncontrolled, destructive growth)
– Hypotrophy (weakened muscle)
– Hypertrophy (increased size and strength of the muscle)

## Systemic arthritis

The TMJ can be affected by various forms of arthritis, including traumatic, infectious, and rheumatoid arthritis (RA) [2]. The origins of RA date back to the 19th century. Although its name was introduced in the 1850s, the classification criteria were developed 50 years ago [4–6]. RA is a chronic, inflammatory, systemic [7], progressive autoimmune [8], erosive, nonsuppurative rheumatic disease with serological evidence of autoreactivity [9] and has an unclear etiology that causes progressive and irreversible joint changes. The disease is characterized by chronic inflammation and synovial hyperplasia, usually affecting multiple joints, with a prevalence of 0.51% in the general population [10]. RA affects not only the elderly but also younger patients [11]. The incidence is higher in women [12], who are affected three times as frequently as men [13]. The onset of the disease occurs mostly between the ages of 40 and 60 years although it may be seen in all ages [14]. In 1874, Garrod described the involvement of TMJ by RA [15]. The development of changes in RA may result in susceptibility of TMJ, but TMJ is rarely affected as the first joint. It is estimated that more than half of the patients with RA present clinical evidence of TMJ involvement [7, 9, 11, 12], with bilateral involvement being the most common [12]. Involvement of TMJ varies greatly from 2 to 88% [16–18]. Genetic factors have a substantial contribution to the development of RA in the population, accounting for 60% of the variation in liability to disease [19]. Smoking is one of the environmental risk factors that double the risk of RA development [20, 21]. Other environmental risk factors include coffee consumption, vitamin D status, alcohol consumption, and poor socioeconomic status although there is no strong supporting evidence with respect to the influence of these factors [22, 23]. RA decreases the quality of patient’s life and eventually prevents them of performing normal daily life routines and professional activities [24].

Chronic pain and joint destruction, which usually progress from distal to more proximal joints, are the main two characteristics of the disease [9]. The involvement of TMJ in patients with RA follows the same destructive path as other joints, correlating directly with the severity and duration of the RA [25]. This is often overlooked, as its clinical manifestations are often silent [26].The most common symptoms of TMJ afflicted with RA may include bilateral pain, joint stiffness, swelling, crepitation, tenderness, difficulties in opening the mouth, limitation of movement, and the open bite, whereas ankylosis is more likely to occur in the late phases of the disease [1, 27]^.^ Whereas ankylosis is more likely to occur in the late phases of the disease [1, 27]. Rheumatoid factor, anticyclic citrullinated antibodies, sedimentation rate, and C-reactive protein are markers of the systemic acute inflammatory response used to diagnose RA [9]. In the initial phase of the disease, there may be signs of synovial hyperemia, lymphocyte infiltration, fibrinoid degeneration, and pannus formation (granulation tissue), followed by the destruction of cartilage and granulation tissue, which can be seen in the joint cavity. From that point on, fibrosis and scarring may occur, generating fibrous adhesion [12]. The central feature of RA is bone destruction. Increased osteoclast activity contributes to local and systemic abnormalities of bone remodeling, including bone erosions and focal and systemic osteoporosis [28]. Cartilage and bone tissue destruction may result in occlusal changes (loss of anterior contacts between the upper and lower jaws) with impaired chewing (function), and if present in children and adolescents, mandibular growth arrest may lead to micrognathia [29–31]. TMJ swelling, redness, or increased temperature occurs very rarely [32], which severely limits the possibilities to identify arthritis of TMJ based on cardinal signs [33]. An anterior open bite is usually a late sign of this disease, but, if there is no pain involved, it could also be the first sign of arthritis of the TMJ. Moreover, it is a clinical sign of ongoing or severe/rapid TMJ cartilage and bone tissue destruction. The incidence of bony deformation in the mandibular condyle was not related to the duration of RA or changes in the other joints [34]. The TMJ is often and early affected by RA [35]. Erosion and flattening of the condylar head, limitation of mobility, and flattening of articular eminentia are the usual radiographic findings in the joint [36, 37]. Meanwhile, soft tissue swelling, periarticular osteoporosis, and loss of interosseous space are considered classic early radiographic manifestations of rheumatic disease. Another important source of pain is a disorder of the muscles of mastication. Patient will often develop muscle tenderness that can be experienced as facial pain if a joint is not functioning correctly [38].

The evaluation of muscle functionality using electromyography in individuals with chronic degenerative diseases is highly valuable, as it highlights changes in the neuromuscular patterns [39]. It is crucial to determine whether these diseases can affect the functioning of the masticatory muscles. RA leads to alterations in muscle function, inflammation of the joint capsule, pain, and resorption of the mandibular head [40]. The balanced functioning of the musculoskeletal system is essential for human health, and functional changes due to chronic degenerative diseases have been extensively studied globally [41]. These changes may be associated with the body’s natural protective response to avoid pain, with edema in the preauricular area reducing the function of the masticatory muscles, resulting in gradual muscle inactivity and motor unit disuse [42]. A lack of local control over proinflammatory cytokines in the joint capsule, which causes pathophysiological and hemodynamic instability and ischemia, is closely linked to decreased masticatory efficiency. This is due to the activation of pain receptors in the nerves during muscle contraction or extension, which increases the production of inflammatory cytokines and leads to a reduction in functional capacity [43]. The pressure that is consciously produced by the contractile force of the lifting muscles of the mandible could be defined as bite force (BF). BF can be identified as a significant predictive factor of disabilities caused by RA. Masticatory muscles activity by electromyography (EMG) has been used in TMJ disorders, orthognathic surgery, and craniomandibular disorders [44–45]. EMG has been widely applied to estimate orofacial muscle function [46]. BF, which results from the action of jaw elevator muscles modified by the craniomandibular biomechanics, is one indicator of the functional state of the masticatory system [47]. The function of masseter and temporalis muscles is changed because of RA on the TMJ [47]. Similar reviews have been conducted in the past with systemic lupus erythematosus [48] but never for RA patients. The aim of this study is thus to review TMJ disorder in RA.

## Methods

The PubMed and Google Scholar databases were searched for articles without restriction on the year of publication. The search covered all available years of publication until March 2024 when the search was completed. Only articles in the English language were taken into consideration. The keywords for search were ‘rheumatoid arthritis’ AND ‘temporomandibular joint’ AND ‘bite forces’ AND ‘masticatory muscles’. Although other databases such as Scopus, Web of Science, and the Cochrane Library could have been included, we focused on PubMed and Google Scholar for their accessibility and wide coverage of medical and dental literature. We acknowledge this as a limitation of our study. This research identified numerous articles, but only seven had a similar aim as our study. These seven articles, along with the reference lists of primary studies, were further searched to identify any additional results. Only articles that were designed as cross-sectional or observational studies and reported masticatory muscle activity in patients with RA in the TMJ were included in this review. Moreover, the articles needed to include data on gender, age, disease duration, diagnosis by a rheumatologist according to the proposed criteria, clinical examination, and laboratory findings. Articles that included exclusion and inclusion criteria for patient selection regarding factors that could impact BFs were specifically preferred. Other articles such as reviews, letters, conference abstracts, editorials, case reports, retrospective, pediatric research, and studies that did not give us information regarding muscle activity in patients with RA in the TMJ were excluded. Additionally, articles included patients with various rheumatic diseases in their studies were not selected for the review.

## Discussion on the main literature

The PubMed and Google Scholar searches for masticatory muscle activity in patients with RA in the TMJ provided seven results. The majority of the searched articles were excluded as they did not meet the inclusion criteria. The abstracts and full texts of each article were analyzed to determine their relevance to the topic. The results of the search on the topic of masticatory muscle activity in patients with RA in the TMJ are summarized in Table 1.

**Table 1:** The list of articles that include the topic of masticatory muscles activity in patients with RA in TMJ.

Reference	Authors and year	Type of study	Inclusion and exclusion criteria	Subjects	CG	Methods	Author’s conclusion
49	Hoyuela et al. 2014	Cross-sectional study	In/age between 18 and 65 years; the presence of teeth with antagonists in the area of the BF measurement; and the presence of teeth or dentures (removable or total) in at least one of the regions of the force measurement. Diagnosis of RA according to the criteria of the American College of Rheumatology without overlap with any other autoimmune disease. The inclusion criteria for the control group included being in good health. Ex/being completely edentulous, using unstable replacement prostheses, having undergone hand or maxillofacial surgery in the previous 6 months, using dental braces and the presence of implants in the region of the force measurement.	75	Yes	- Clinical examination; - BF measurement with dynamometer; - The Oral Health Impact Profile (OHIP-14) questionnaire to evaluate the quality of life related to oral health, - HS	Women with RA present more signs and symptoms in the orofacial region and have a lower BF than the control group. Bite force was inversely correlated with the overall function (evaluated by the HAQ) in these patients, and there was a correlation between BF and HS in the RA patients and in the healthy control group.
50	Larheim et al. 1985	Cross-sectional study	In/patients with natural teeth and abnormalities of the TMJ, the mean age of the patients was 51 years with a range from 25 to 74. The mean duration of RA was 17 years (range 4–28).	16	Yes	- Physical examination, - BF measurement using a miniature bite force sensor, - The lateral transcranial radiographs at intercuspal position and at maximal mouth opening.	Maximal bite force in the RA group was significantly lower than in the control group. The rheumatic patients bit with nearly the same load as the healthysubjects did when chewing.
51	Palinkas et al. 2018	Observational study	In/women with RA diagnosed by rheumatologists, based on the clinical signs, symptoms, and laboratory and radiographic findings, with a median disease duration of six years, and with an uncontrolled disease in the past year Ex/women with bruxism in wakefulness and in sleep, previous obstructive sleep apnea diagnosis, mandibular tori, current or previous orthodontic treatment, physical therapy, physical changes (from infection, trauma, accident, intoxication) or mental illness, neurological and psychiatric disorders, and use of medications (muscular relaxant, antihistamines, sedatives, or central nervous system depressors) that could interfere with the electromyographic activity of the skeletal striated muscles.	14 (women)	Yes	- EMG (for masseter and temporalis muscles). - BF with paraffin sheet.	Women with rheumatoid arthritis showed functional alterations in the stomatognathic system, demonstrated through muscular hyperactivity and reduction of masticatory efficiency.
52	Hiz et al. 2012	Cross-sectional study	In/patients diagnosed with RA at least 6 months before and received DMARD + steroidal/nonsteroidal treatment. Ex/patients who had jaw-related traumas, with teeth and gum diseases to the extent that it affects jaw force, total or partial upper or lower dentures, bruxism history, facial nerve paralysis, cerebrovascular event, trigeminal neuralgia, and polyneuropathy history, active psychiatric disease history, and diseases with juvenile onset and who underwent TMJ injection in the last 6 months.	30	Yes	- Clinical examination; - BF measurement with dynamometer; MRI, - DAS 28 score, - Fonseca questionnaire, - Lab (CRP, ERS, RF).	Jaw clenching force, mouth opening, and Fonseca’s questionnaire can be used as parameters pointing to TMJ involvement in patients with RA. Yet, further studies in which TMJ involvement is followed up since the onset of the disease is of necessity.
53	Cruz-Hervert et al. 2017	Cross-sectional study	In/18 years of age or older at the initial record time and with no evidence of craniofacial abnormalities. Ex/patients with incomplete records and with severe temporomandibular disorders or acute orofacial pain.	60	Yes	- Clinical examination - EMG (for masseter muscle)	EMG in patients with rheumatoid arthritis had higher mean values of RMS but a decreased activity over the evaluated period. More research is needed to clarify the electromyographic activity in patients with rheumatoid arthritis.
54	Rodrigues et al. 2017	Cross-sectional study	In/women with RA with a median disease duration of six years with the clinical signs, symptoms, and laboratory and radiographic findings, and with an uncontrolled disease in the past year. Ex/women with bruxism, bruxism in wakefulness, obstructive sleep apnea syndrome, mandibular tori, neurological or psychiatric disorders, physical or mental illness, who were under physiotherapeutic treatment and in the use of medications that may interfere with skeletal muscle activity.	14 (women)	Yes	- Measurement the thickness of the masseter and temporalis muscles by US. - BF measurement with dynamometer - The OHIP-14 questionnaire - DAS 28. - Lab findings (ACCP, RF, ERS). - CT - Mandibular mobility.	Women with rheumatoid arthritis showed decrease in maximum molar bite force and negative impact on the repercussion of oral conditions on the quality of life. However, there was no significant statistical difference for masticatory muscles’ thickness and mandibular mobility
55	Yilmaz et al. 2012	Cross-sectional study	Ex/subjects that cannot undergo MRI	28	Yes	- Clinical examination - DAS 28 score - Lab findings (ESR, CRP, RF). - Panoramic radiographs (OPGs), lateral panoramic radiographs in open and closed mouth positions. - MRI to evaluate TMJ and masticatory muscle thickness and area.	TMJ symptoms were thought to be affected from the mean disease duration time in RA. Also, laboratory findings should be considered for disease activity and related TMJ involvement. The results of muscle thickness and area measurements in our study did not suggest muscular atrophy or hypertrophy in RA patients when compared with control group. Clinical and radiological follow-up of RA patients with TMJ and masticatory muscle complaints is essential.

In: inclusion; Ex: exclusion; CG: control group; RA: rheumatoid arthritis; BF: bite force; TMJ: temporomandibular joint; HS: hand strength; EMG: electromyography; OHIP-14: Oral Health Impact Profile Questionnaire; ESR: sedimentation rate; CRP: C-Reactive protein; RF: rheumatoid factor; MRI: magnetic resonance imaging; RMS: root medium square; US: ultrasound; CT: computed tomography.

In a 2014 cross-sectional analysis, Hoyuela and colleagues [49] examined 150 women, including 75 diagnosed with RA at a specialized outpatient clinic. The study required participants to be aged 18 to 65 years, possess opposing teeth for BF measurements, and have at least one region with teeth or dentures for force assessment. The research also included 75 healthy individuals as a control group. Exclusions were made for those without any teeth, unstable prostheses, recent maxillofacial surgery, dental braces, or implants in the force measurement area. Both RA and control groups were comparable in age, gender, and socioeconomic status. RA patients were assessed by a rheumatologist using the DAS-28 index (Disease Activity Score in 28 joints) just before joining the study. BF was gauged bilaterally on molars and incisors using a calibrated dynamometer. The Oral Health Impact Profile (OHIP-14) questionnaire assessed oral health-related quality of life, and a physiotherapist conducted the hand strength evaluation. The findings indicated that RA patients exhibited more orofacial signs and symptoms and a reduced BF compared to controls. There was an inverse correlation between BF and overall function (assessed by the Health Assessment Questionnaire) in RA patients and a link between BF and hand strength in both RA and healthy groups.

In 1985, Larheim and team [50] conducted a cross-sectional study on 16 individuals (14 women and 2 men) diagnosed with RA, focusing on those with natural teeth and TMJ abnormalities. The average age was 51, ranging from 25 to 74, and the average RA duration was 17 years (ranging from 4 to 28 years). The study also evaluated maximal mouth opening, translatory condylar motion, and BF in 16 healthy individuals without joint disease, serving as controls. These controls had no TMJ symptoms or radiographic issues. Following physical exams and lateral tomograms, the researchers found that the RA group’s maximal BF was significantly lower than that of the control group although the RA patients exerted nearly the same force as healthy subjects during chewing.

Palinkas and associates [51], in 2018, aimed to assess the EMG activity of the masseter and temporalis muscles in women with RA through an observational study. The sample included 28 women, half with RA and half without. Participants were matched by age and body mass index. The study utilized electromyography for the masseter and temporalis muscles, with paraffin sheets used for dental clenching in maximum voluntary contraction. The results revealed functional changes in the stomatognathic system of RA patients, characterized by muscular hyperactivity and decreased masticatory efficiency.

Hiz and colleagues [52] in 2012 investigated TMJ involvement in RA patients. The study included 30 RA patients and 30 healthy volunteers, excluding those with trauma, significant teeth and gum diseases, dentures, bruxism, facial nerve paralysis, cerebrovascular events, trigeminal neuralgia, polyneuropathy, active psychiatric diseases, juvenile-onset diseases, or recent TMJ injections. Alongside clinical exams, DAS 28 scores, and laboratory tests (C-reactive protein [CRP], sedimentation rate [ESR], and RF), magnetic resonance imaging (MRI), Fonseca’s questionnaire, and a BF measurement device were used. The study concluded that jaw clenching force, mouth opening, and responses to Fonseca’s questionnaire could indicate TMJ involvement in RA patients, suggesting the need for further research on this topic.

In 2017, Cruz-Hervert et al. [53] performed a cross-sectional study on 60 RA patients to evaluate EMG activity in those with and without RA. The study involved clinical examinations, sociodemographic data collection, and surface EMG for the masseter muscles. Participants were adults with no craniofacial abnormalities. Exclusions were made for incomplete records and severe temporomandibular disorders or acute orofacial pain. The findings showed higher EMG activity in RA patients, who also experienced a greater reduction in muscle activity due to fatigue. The authors recommended more comprehensive research to support these findings.

Rodrigues and colleagues [54] in 2017 conducted a cross-sectional study to analyze 14 adult women with early-stage RA, without therapeutic control for over a year, and with complete dentition, comparing them to 14 healthy women. The study measured masticatory muscle thickness, BF, mandibular mobility, and the impact of oral conditions on the quality of life. Participants were excluded if they had bruxism, sleep apnea, and neurological or psychiatric disorders or were undergoing treatments that could affect muscle activity. The results indicated that women with RA had reduced BF and a negative impact on their oral health-related quality of life.

Lastly, Yilmaz and associates [55] conducted a cross-sectional study with 28 RA patients and 29 healthy matched subjects to determine changes in masticatory muscles and TMJ in RA patients. The average disease duration was 5 years, and those unable to undergo maxillofacial MRI were excluded. The MRI evaluations showed no significant muscular atrophy or hypertrophy in RA patients compared to the control group.

Based on our review, patients with RA exhibit significant functional alterations of the masticatory muscles, characterized by reduced BF and changes in EMG activity. Although most studies confirmed reduced occlusal force, the degree of reduction varied and was not directly correlated with the progression of RA due to the cross-sectional design of the available studies. Longitudinal research is necessary to quantify the exact rate of muscle activity decline and its relation to RA severity.

A multidisciplinary diagnostic protocol should be considered for patients with suspected TMJ involvement in RA. This could include: clinical examination of pain, stiffness, and jaw mobility; electromyography to assess masticatory muscle activity; BF measurements; imaging methods such as cone-beam computed tomography (CBCT) and MRI for detecting bone and soft tissue changes; and rheumatologic markers such as CRP, ESR, RF, and anti-citrullinated protein antibodies (ACPA) to correlate systemic disease activity with TMJ findings. Such a protocol would allow earlier detection and more comprehensive management of TMJ disorders in RA patients.

While TMJ symptoms alone are not sufficient to diagnose RA, their presence should raise clinical suspicion and encourage referral to a rheumatologist. This is particularly relevant when patients present with unexplained jaw pain, stiffness, or reduced BF.

## Limitations of the study

RA as a systemic etiological factor has a major impact on the development of temporomandibular disorders. The effects that RA has on masticatory muscles and surrounding tissues, call for more-much needed studies. Thus, research on the effects of RA on the stomatognathic system is expected to increase based on the fact that this field has not been extensively investigated in the past. With the aim of determining the BF in the masticatory muscles and TMJ in RA patients, we intended to evaluate all available evidence pertaining to the function of major chewing muscles. To the best of our knowledge, the masticatory BF in patients with RA in the TMJ has not been evaluated. This could be explained by the fact that physicians may have less interest in the TMJ in RA patients during daily practice, which could lead to overlooking TMJ involvement at the time of questioning the patient. Our study had limitations. The main limitation of the current review is the small number of eligible studies (*n* = 7). Furthermore, all of the included studies were cross- sectional, preventing assessment of disease progression over time. As a result, we cannot provide precise estimates of the rate of TMJ disorder progression in relation to RA progression. The heterogeneity of study methodologies, including different BF measurement devices and inconsistent inclusion/exclusion criteria, also limited comparability. Additionally, the search was limited to PubMed and Google Scholar databases, which may have excluded relevant studies indexed elsewhere. Some of the studies did not have strict limitations on patients’ age and gender, and others did not match pairs, so only the most relevant studies were addressed here. Regarding the selection of the subjects and inclusion and exclusion criteria, they were not consistent across all investigations. Despite having the same objective, the BF has been measured by different devices.

## Final analysis and future perspectives

Given the limited yet growing research on masticatory muscle activity in patients with RA involving TMJ, several future directions are recommended. Longitudinal studies are needed to monitor TMJ and masticatory muscle changes over time, particularly in relation to RA activity and treatment response; advanced imaging techniques such as high-resolution MRI and ultrasonography should be applied to detect early structural and functional changes; clinical trials should be designed to evaluate TMJ rehabilitation protocols, including physiotherapy and targeted dental interventions, to preserve muscle function and prevent irreversible complications; the effect of novel therapies, including biologic agents, on TMJ and masticatory muscle function should be investigated; greater emphasis should be placed on patient-reported outcomes, addressing the impact of TMJ dysfunction on daily activities, psychosocial well-being, and quality of life. Exploring genetic and molecular mechanisms underlying TMJ involvement in RA could also provide biomarkers for earlier diagnosis and targeted therapies. This may help establish preventive strategies and tailored treatment plans to protect oral health and overall patient well-being.

Most currently existing studies are cross-sectional, providing only a snapshot of muscle function at a single point in time. Future research should focus on tracking changes in masticatory muscle activity over time in RA patients, particularly in relation to disease progression and treatment interventions. This would offer more comprehensive insights into the temporal dynamics of muscle involvement in RA. Current studies often involve small, homogenous populations, predominantly women. Future research should include larger and more diverse populations, incorporating a broader age range and varying stages of RA. While EMG and BF measurements are commonly used, future studies could incorporate more advanced imaging techniques like high-resolution MRI or ultrasonography. These tools could provide more detailed assessments of muscle structure and function, allowing for earlier detection of subtle changes in the masticatory muscles and TMJ. With the advent of biologic agents and other novel therapies for RA, future studies should investigate how these treatments impact masticatory muscle function and TMJ involvement. Understanding the therapeutic effects on these specific areas could inform more personalized treatment plans aimed at preserving oral health and function in RA patients. While some studies have assessed quality of life related to oral health, more research is needed to explore patient-reported outcomes in greater detail. This includes understanding how changes in masticatory muscle function affect daily activities, psychosocial well-being, and overall quality of life in RA patients. Given the evidence of reduced BF and masticatory efficiency in RA patients, future research should also focus on developing preventive measures and rehabilitation strategies. This could involve designing specific physical therapy exercises or dental interventions to maintain or restore muscle function and TMJ health. Finally, exploring the genetic and molecular mechanisms underlying masticatory muscle involvement in RA could provide new insights into disease pathology. Identifying biomarkers associated with muscle dysfunction could lead to earlier diagnosis and more targeted treatments, potentially preventing severe TMJ complications.

## Conclusions

RA of TMJ is a chronic condition that significantly impacts quality of life by impairing chewing, speech, and orofacial function. The studies reviewed indicate that RA of the TMJ leads to reduced masticatory muscle activity, limited mouth opening, and orofacial pain. Given these findings, we propose incorporating TMJ evaluation into the routine comprehensive assessment of RA patients. Early detection and interdisciplinary management involving rheumatologists, maxillofacial surgeons, and dentists are essential. Future studies should focus on developing standardized diagnostic protocols, rehabilitation strategies, and preventive measures aimed at preserving masticatory muscle function and improving the quality of life for RA patients.
